# Accelerated decline in cardiac stem cell efficiency in Spontaneously hypertensive rat compared to normotensive Wistar rat

**DOI:** 10.1371/journal.pone.0189129

**Published:** 2017-12-12

**Authors:** Sherin Saheera, Renuka R. Nair

**Affiliations:** Division of Cellular and Molecular Cardiology, Sree Chitra Tirunal Institute for Medical Sciences and Technology, Trivandrum, Thiruvananthapuram, Kerala, India; Texas A&M University Health Sciences Center, UNITED STATES

## Abstract

Cardiac hypertrophy is recognized as an independent risk factor for cardiac failure. Efficient management of hypertensive heart disease requires identification of factors that can possibly mediate the transition from hypertrophy to failure. Resident cardiac stem cells have a prominent role in the maintenance of cardiac tissue homeostasis. Decline in the proportion of healthy cardiac stem cells (CSCs) can affect tissue regeneration. In pathological conditions, apart from natural aging, an adverse microenvironment can lead to decrease in efficiency of CSCs. A systematic analysis of cardiac stem cell characteristics in pathological conditions has not been reported so far. Therefore, this study was designed with the objective of examining the age associated variation in stem cell attributes of Spontaneously hypertensive rat (SHR) in comparison with normotensive Wistar rat. Spontaneously hypertensive rat was used as the experimental model since the cardiac remodeling resembles the clinical course of hypertensive heart disease. CSCs were isolated from atrial explants. Stem cell attributes were assessed in 1-week, 6, 12 and 18-month-old male SHR, in comparison with age matched Wistar rats. In 1-week-old pups, stem cell attributes of SHR and Wistar were comparable. Migration potential, proliferative capacity, TERT expression, telomerase activity and the proportion of c-kit^+^ cells decreased with age, both in SHR and Wistar. DNA damage and the proportion of senescent CSCs increased with age both in SHR and Wistar rats. Age associated increase was observed in the oxidative stress of stem cells, possibly mediated by the enhanced oxidative stress in the microenvironment. The changes were more pronounced in SHR, and as early as six months of age, there was significant decrease in efficiency of CSCs of SHR compared to Wistar. The density of healthy CSCs determined as a fraction of the differentiated cells was remarkably low in 18-month-old SHR. Age associated decrease in functionally efficient CSCs was therefore accelerated in SHR. Considering the vital role of CSCs in the maintenance of a healthy myocardium, decrease in functionally efficient CSCs can be a precipitating factor in pathological cardiac remodeling. Elevated ROS levels in CSCs of SHR lends scope for speculation that decrease in efficiency of CSCs is mediated by oxidative stress; and that modulation of the microenvironment by therapeutic interventions can restore a healthy stem cell population and facilitate maintenance of cardiac homeostasis and prevent cardiac decompensation.

## Introduction

Left ventricular hypertrophy (LVH) remains a powerful indicator of impending cardiac failure.

[1] The cause for the progression from compensatory phase of left ventricular hypertrophy to decompensatory phase remains enigmatic. The heart was considered to be a terminally differentiated organ, without capacity for tissue repair and regeneration. Identification of resident cardiac stem cells (CSCs) contradicted the paradigm that the myocardium is a post-mitotic organ. In human hearts there is 0.5 to 1% of myocyte turnover annually,[2] envisaging the role of CSCs in the maintenance of cardiac tissue homeostasis. CSCs differentiate and replace the lost myocytes; and in the event of myocardial injury, stem cells contribute towards tissue repair.[3,4] The involvement of stem cells in cardiac failure associated with age and disease has been speculated.[5,6] However, the temporal variation in the density and efficiency of cardiac stem cells and the effect of disease on the stem cell characteristics has not been systematically analyzed. There is only one report, where Cesselli et al examined the cardiac stem cells from failing hearts of patients undergoing cardiac transplantation in comparison with donor hearts and inferred that efficiency of cardiac stem cell deteriorates with age and cardiovascular disease. [7] However, lack of appropriate age and disease matched control precluded a confirmatory statement on the distinction between pathological and physiological aging of CSCs.[7] Nakamura et al observed a good correlation with age in the expression of senescence markers in cardiosphere derived cells from aged hearts; but, no correlation was observed between age and growth rate, angiogenic ability and growth factor production.[8] These preliminary observations in human samples underscore the requirement for a systematic analysis of the variation in stem cell characteristics with age and disease, using an appropriate animal model. Deterioration in stem cell characteristics is possibly mediated by a suboptimal microenvironment. LVH is associated with myocyte loss.[9] Oxidative stress increases both with age and cardiac disease.[10,11] Myocardial oxidative stress is implicated in pathological cardiac remodeling.[12–14] Increased oxidative stress in the surrounding milieu can influence the stem cell characteristics [15,16]. Therefore, stem cell number and efficiency can decrease with age, and with greater intensity in pathological conditions. The present study was carried out based on the premise that, “The functional efficacy of resident cardiac stem cells decrease with age and at an accelerated rate in Spontaneously hypertensive rat.” The study was designed with the objective of examining the density and functional efficacy of cardiac stem cells in the pathological heart in comparison with the normal heart. In view of the decrease in cardiac efficiency as a function of age, the temporal variation in stem cell characteristics was assessed in normotensive Wistar rat (WST) and compared with the changes in Spontaneously hypertensive rat (SHR). The density, proliferation efficiency, migration potential, senescence and DNA damage profiles as well as oxidative stress of CSCs at different stages of cardiac remodeling were assessed.

## Methods

### Design of study

The study was designed with the objective of examining the age-associated changes is stem cell characteristics of SHR in comparison with WST rat. SHR is a genetic model of hypertension, where the cardiac changes are similar to that seen in clinical hypertension [17] with transition from compensated ventricular hypertrophy to failure.[18] Male SHR of different ages- pups (1-week old), 6-months, 12-months and 18-months were selected representing various stages of cardiac remodelling. No significant cardiac changes occur before 1 month of age. Six months represents the stable phase of hypertrophy. Cardiac decompensation is initiated at 12 months and established by 18 months of age.[19,20] Studies using aged SHR has shown typical features of heart failure reiterating the appropriateness of this model.[21,22] Oxidative stress is apparent from 1 month of age and precedes hypertrophy.[23] The percentage of CSCs was determined from ventricular digests, as a fraction of the myocardial cells. Migration, proliferation, differentiation and senescence were evaluated in CSCs isolated from atrial explants. Three animals were analysed in each group.

The study was approved by The Institutional Animal Ethics Committee. The housing care and the management of these animals were in accordance with the Committee for the Purpose of Control and Supervision of Experiments in Animals (CPCSEA) Guidelines.

### Experimental methods

#### Maintenance of animals

Spontaneously hypertensive rat were purchased from Animal Resource Center, Perth, Australia; and maintained in the Division of Laboratory Animal Science of the Institute. The animals were housed at 22±2°C and 55±10% RH in individually ventilated cages. Light levels measured at 1 meter height were less than 300 Lux and a 12:12 hour dark: light pattern was maintained. The animals were fed with standard rat pellet feed and drinking water *ad libitum*. Experimental animals were euthanized under deep surgical plane of anesthesia using 5mg/kg Xylazine and 70mg/kg Ketamine followed by 10mg/kg body weight of 1% Thiopentone sodium as i/p injections. The heart was immediately dissected out and the ventricles were separated from the atrium.

### Determination of fraction of c-kit^+^ cells in the ventricle

Ventricular tissue was enzymatically digested using collagenase following standard protocol.[24] The ventricular tissue was minced into 2–3mm size cubes and digested in collagenase for 15 minutes. The suspension of dissociated cells was separated out and the enzyme was inactivated using serum containing medium. The undigested tissue was treated with collagenase. The digestion time was kept constant to prevent cell damage. The digestion process was repeated till the ventricular tissue was completely dissociated. The tissues from older rat required more number of repeated digestions. The proportion of cardiac stem cells was determined as ratio of differentiated cells. The cells from the different digests were pooled and suspended in phosphate-buffered saline (PBS) and the density of c-kit^+^ CSCs was determined by flow cytometry analysis. The percentage of ckit^+^ cells was obtained as a fraction from 10000 ventricular cells.

### Isolation and expansion of CSCs from atrial explants

To obtain cells in sufficient numbers for evaluation of stem cell attributes, CSCs were isolated from atrial explants and expanded in culture following established protocol.[3] Phase-bright cells were seen loosely attached over a layer of fibroblast like cells when atrial explants were cultured for 2 weeks (S1 Fig). C-kit^+^ cells were isolated from the cellular outgrowths using EasySep^TM^ magnet and FITC positive selection kit; and expanded in culture in IMDM containing 10% FBS, 10 ng/mL bFGF and 10μL/mL insulin-selenium-transferrin mixture at 37°C with 5% CO_2_. (S1 Fig).

The cells were characterized for cell surface markers: c-kit, CD31 and CD45 by immunostaining and flow cytometry analysis. For immunostaining, the cells were incubated overnight at 4°C with the primary antibody followed by incubation with FITC-conjugated secondary IgG at 25°C for 1h and counterstained with 1μL/mL Hoescht. The immunostained cells were examined using fluorescent optics. Flow Cytometric Analysis of single cell suspension was carried out using FACS Cabilur with Cell Quest software (BD Biosciences).[25] Flow cytometric analysis and immunocytochemistry of the cells collected at passage 3 showed that 95.5±10% were positive for c-kit and negative for both CD31 and CD45 (S1 Fig). Retention of cellular identity despite repeated subculture was apparent from the observation that 74±6% of the cells was c-kit^+^ even at passage 10 and none expressed CD45 and CD31 (S1 Fig). Stemness was further confirmed by clonogenic assay.

For clonogenic assay, CSCs in the 3^rd^ passage were harvested by trypsinization, serially diluted and seeded into a 96-well plate at a density of 0.5 cell per well, to obtain single cell clones.[26] After 2 weeks, the number of wells with clones derived from a single cell was counted. Clonogenicity was determined using the following formula:

Clonal efficiency (%) = (Number of wells with clones/Total wells with single cell) x100.

Colony formation was apparent at all ages. On an average, 90±6% of single cells of CSCs formed colonies.

### Assessment of characteristics of CSCs isolated from atrial explants of SHR and Wistar rat

#### Growth kinetics

A total of 10000 c-kit^+^ CSCs from passage 3 was cultivated in 35 mm dishes for a period of 10 days. Cells were detached by trypsinisation and numbers were determined every 48 h using a Neubauer haemocytometer. Growth rate (GR) and population doubling time (PDT) were mathematically calculated. GR = ln(N_t_/N_0_)/T, where T is the incubation time, N_0_ is the cell number at the beginning of the incubation time and N_t_ is the cell number at the end of the incubation time. Population doubling time was calculated using the formula, PDT = ln(2)/GR.

#### Cell migration

Cell migration was analyzed by trans-well migration assay. CSCs were suspended in serum free medium and 10000 cells were seeded on to the upper chamber of trans-well (pore size—8μm). Serum containing medium was used as the chemo-attractant and placed in the lower chamber. Following incubation for 12 h at 37°C, the cells that migrated to the lower surface of the membrane was fixed, stained and counted using a microscope.

#### Colony forming unit assay

The cells were seeded at a density of 500 cells per 60mm diameter culture plate. Growth media was changed once in 3days. After 11 days, the cells were stained with crystal violet and colonies larger than 2mm were counted.

#### Senescence assay

Cellular senescence was assessed by cytochemical analysis of senescence-associated β-galactosidase staining using a commercially available kit (Abcam). A minimum of 100 cells from 5 randomly selected areas were counted for each culture and positively stained cells were expressed as percentage of senescent cells.

The expression of senescence associated marker proteins p16^ink4a^ and p21 were assessed in cellular extracts by western blot analysis. Enhanced chemiluminescence reagent was used to visualize the proteins, and protein expression was quantified by densitometry scanning.

#### Detection of ROS levels in CSCs

ROS levels of CSCs was measured based on the oxidation of 2’,7’-dichlorodihydrofluorescein (DCFH). Following incubation with the fluorescent dye for 10 minutes, fluorescence intensity of the cultured cells was measured at excitation and emission wavelengths of 498 nm 530 nm respectively.

#### Cell differentiation assay

Cell differentiation was stimulated by exposing the cells to 5-azacytidine followed by incubation for 3 weeks. Cellular protein was extracted and expression of cell-specific markers cardiac troponin I (cTnI) and smooth muscle actin (SMA) were examined by western blot analysis using β-actin as loading control.

#### Telomerase activity

Telomerase activity in cells was detected by the modified TRAP assay using the TRAPeze Telomerase Detection Kit (Millipore, S7700). The assay is a one-buffer, two-enzyme system using the PCR. In the first step of the assay, telomerase adds the 6-bp telomeric sequence (TTAGGG) onto the 3' end of a 5' end infrared dye–labeled oligonucleotide substrate (5'-AATCCGTCGAGCAGAGTT- 3') (TS Primer,Kit). In the second step, the extended products are amplified by PCR. Briefly, 10^6^ cells of each sample were resuspended in 200μL of 3-[(3-cholamidopropyl)dimethyl-ammonio]-1- propanesulfonate (CHAPS) lysis buffer (Kit) and incubated for 30 min on ice. After the incubation, lysates were centrifuged at 12,000 *g* for 20 min at 4°C. The supernatant was recovered, and the protein concentration was measured using the Biciuchoriuic acid (BCA) test. Because the best differentiation of the samples was seen with extracts containing 500ng protein, this concentration was used as the standard concentration. To heat inactivate the telomerase 5μl of each sample extract was incubated at 85°C for 10 min. TSR8 and CHAPS buffer (Kit) were used as positive and negative controls, respectively. After a 30-min incubation at 30°C, the samples were subjected to 33 PCR cycles of 94°C for 30 s, 58.3°C for 30 s and 72°C for 1 min. The PCR products were separated by electrophoresis on non denaturing polyacrylamide gels. The gels were stained with Ethidium Bromide and viewed under a trans-illuminator and photographed. Telomerase activity on digital images was quantified using ImageJ software, (Version 1.42q—NIH) as units of total product generated (TPG), based on the formula: TPG = [(*x—x*_o_)/*c* x100]/[(*r—r*_o_)/*c*R], where *x* is the signal of the region of the gel lane corresponding to the TRAP product ladder bands from non-heat-treated samples, *x*_o_ is the signal of the region of the gel lane corresponding to the TRAP product ladder bands from heat-treated samples, *r* is the signal of the region of the gel lane corresponding to the TRAP product ladder bands from TSR8 quantitation control, *r*_o_ is the signal of the region of the gel lane corresponding to the TRAP product ladder bands from 1X CHAPS lysis buffer-only control, *c* is the signal from the internal standard (S-IC) in non-heat-treated samples, and *c*R is the signal from the internal standard (S-IC) in TSR8 quantitation control. Each unit of TPG corresponds to the number of TS primers extended with at least 4 telomeric repeats by telomerase in the extract in a 30 min incubation at 30°C.

#### Expression of TERT by reverse-transcriptase PCR

RNA was extracted from CSCs and transcribed to cDNA using commercially available kits (Qiagen, TAKARA). The real-time PCR reaction mixture consisted of cDNA, SYBR green, forward and reverse primers for a final reaction volume of 20μl. 18SrRNA was used as loading control (Table 1). The threshold cycle (CT) for fluorescence development was calculated as fold change using the formula “2^-ΔΔCT^ method.” All PCR reactions were performed using the ABI Prism 7500 Sequence Detection System (Applied Biosystems).

**Table 1:** Sequence of Oligionucleotide primers.

Gene name	Forward Primer	Reverse primer
*TERT*	5’AGTGGTGAACTTCCCTGTGG 3’	5’CAACCGCAAGACTGACAAGA3’
*18SrRNA*	5’TCAAGAACGAAAGTCGGAGG3’	5’GGACATCTAAGGGCATCAC3’

#### Comet assay

Comet assay was performed as described by Hermeto et al.[27] Comets were visualised using the fluorescent dye ethidium bromide. Images were captured using an intensified solid state CCD camera attached to the microscope and linked to the Comet Assay II image analysis software. Samples were run in duplicate, and 50 cells were randomly analyzed per slide for a total of 100 cells per sample. The comet evaluation was carried out based on the tail moment.

### Statistical analysis

Values are expressed as Mean±SD. Two way ANOVA was carried out and in the event of significant variation this was followed by two-tailed unpaired Student’s *t*-test for comparison of SHR and WST rat. Results were considered statistically significant for p<0.05.

## Results

### Proportion of stem cells in the ventricular myocardium

**Fig 1:**
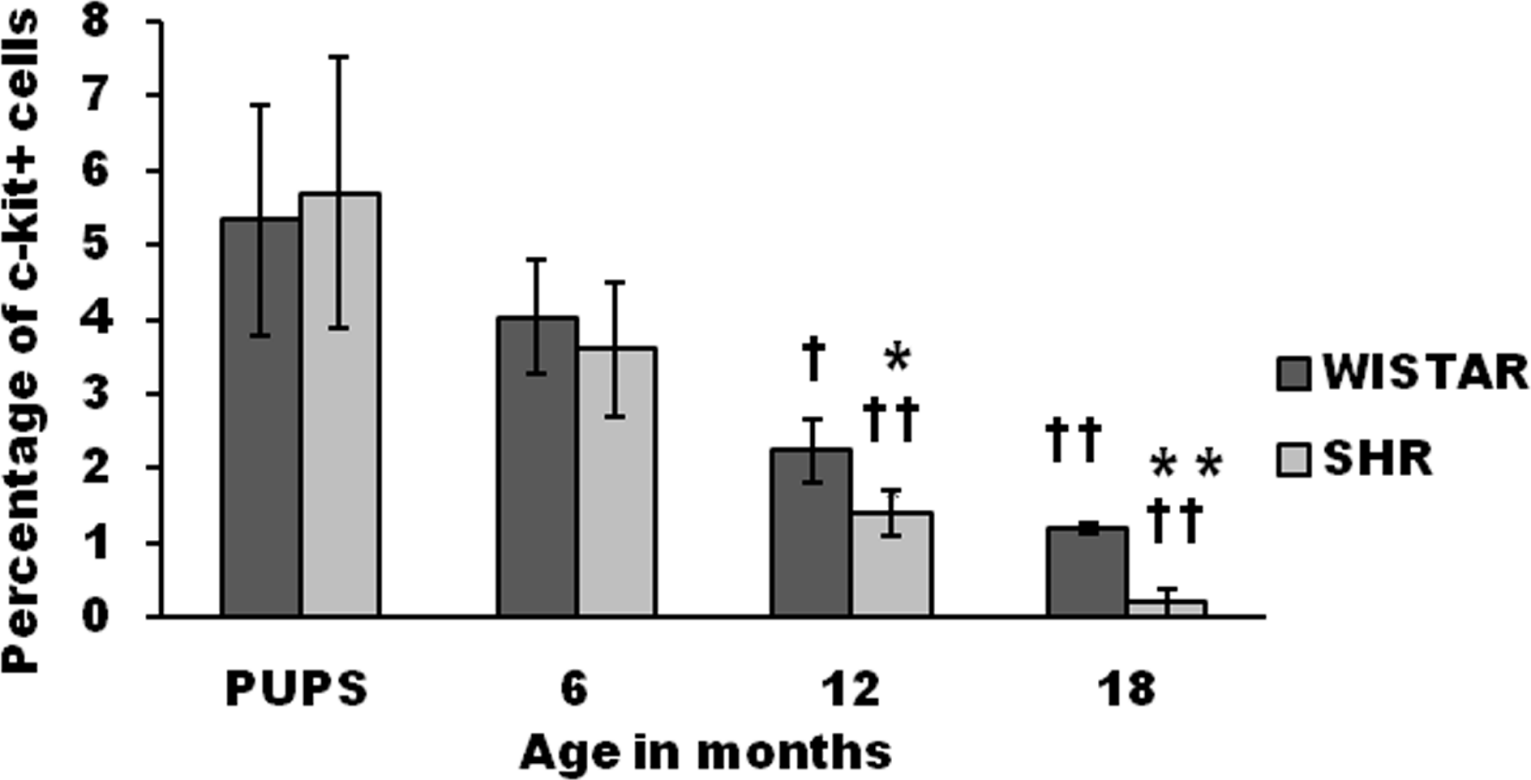
Age associated variation in the proportion of c-kit^+^ CSCs in ventricular digests from SHR and Wistar rats. Graphical representation of data expressed as percentage of total cells. (n = 3). Data presented as mean ± SD. Variation was analysed by two way ANOVA followed by Student t-test. (** p<0.01 and * p<0.05 SHR Vs Age matched WST; †† p<0.01 and † p<0.05 WST & SHR of different ages compared to respective 1 week old pups. Two way ANOVA p<0.001).

The proportion of c-kit^+^ stem cells in ventricular digests were determined by FACS analysis. The proportion of stem cells decreased with age in both SHR and WST rat (Fig 1). Age associated decline in CSCs, compared to one-week old pups was statistically significant at 12 months and 18 months. The density of CSCs in 1 week old pups and 6 month old SHR and Wistar rats was not significantly different. At 12 months, a significant decrease was observed in SHR. By 18 months the proportion of c-kit^+^ cells declined further, where the proportion in SHR was as low as 1/20 of the value in pups compared to 1/4 in Wistar^.^ of that in pups in Wistar rat.

### Self-renewal capacity of cultured CSCs

**Fig 2:**
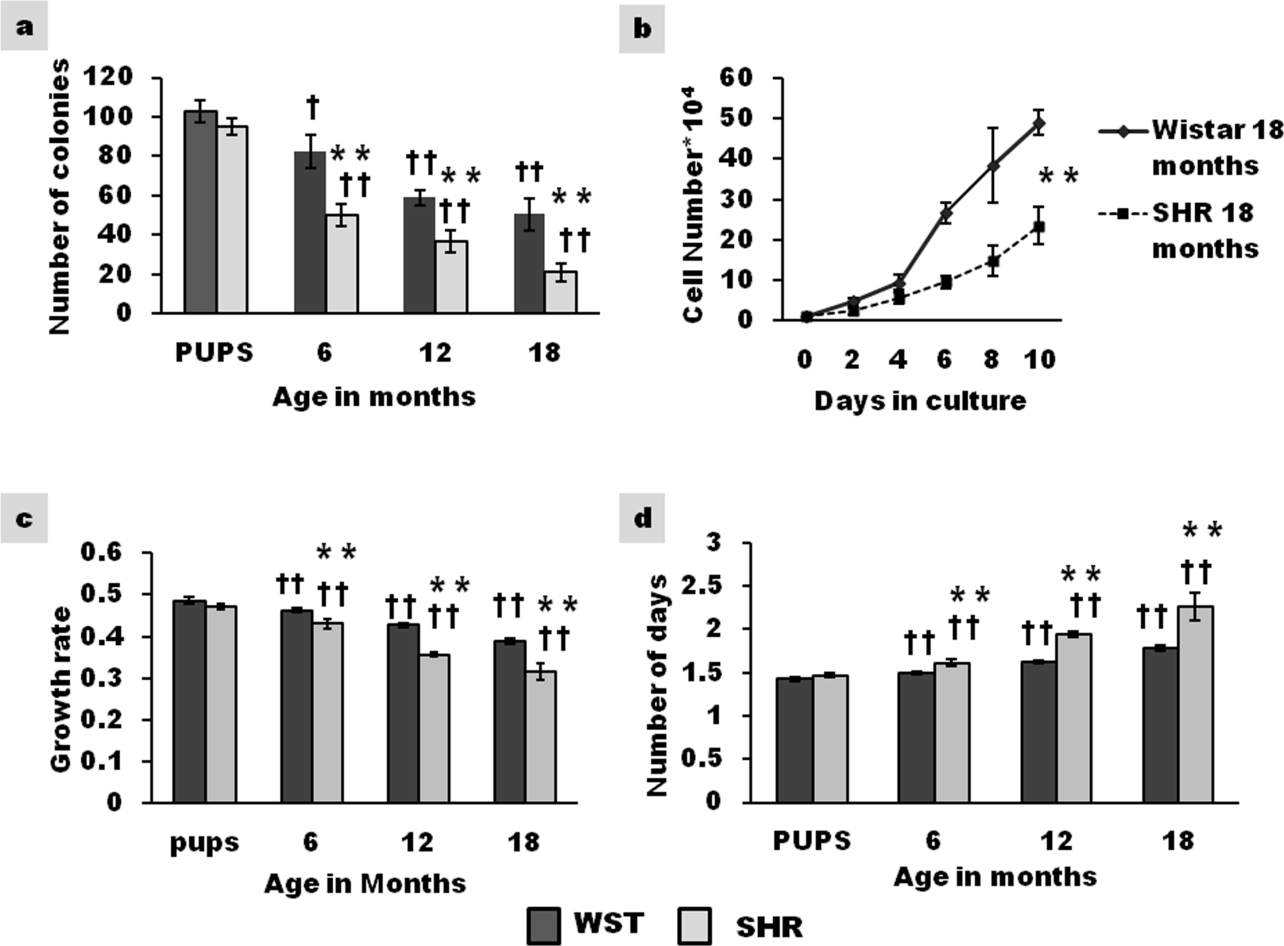
Age associated variation in growth characteristics of CSCs cultured from SHR and Wistar rats. **(a)** Colony Forming Units representing number of colonies formed (n = 3) **(b)** Growth kinetics of 18-month old rats represented as cell number*10^4^ (n = 3) **(c)** Growth rate was calculated as LogN of the ratio of cell number at two fixed time points (n = 3) **(d)** Population doubling time (PDT) represented as number of days (n = 3) Data presented as mean ± SD. Variation was analysed by two way ANOVA followed by Student t-test. (** p<0.01 SHR Vs Age matched WST; †† p<0.01 and † p<0.05 WST & SHR of different ages compared to respective 1 week old pups. Two way ANOVA p<0.001).

Colony forming unit is a variable used for evaluating stemness. Aging had a visible effect on colony forming ability. The number of CFUs was comparable in 1 week old pups of both the strains. Compared to that in pups, at 18 months of age, the number of colonies formed in WST was 50% and as low as 25% in SHR (Fig 2A). The size of the colonies was larger in Wistar rat compared to SHR at all ages. These findings possibly indicate age associated decline in stemness, with the decrease being accelerated in SHR.

### Temporal variation in growth kinetics, population doubling time (PDT) and growth rate of CSCs in culture

Following 10 days in culture, the yield of CSCs from 18-month-old SHR (23.52±4.5*10^4^) was half of that found in age matched WST rat (49±3*10^4^) (Fig 2B). Age associated decrease in growth rate (Fig 2C) with consequent increase in population doubling time (Fig 2D) was observed, the decline being significantly higher for SHR. Compared to that in pups, CSCs from 18 month old WST and SHR exhibited an increase in PDT by 7 hrs and 20 hrs respectively.

### Migration efficiency of cultured CSCs

**Fig 3:**
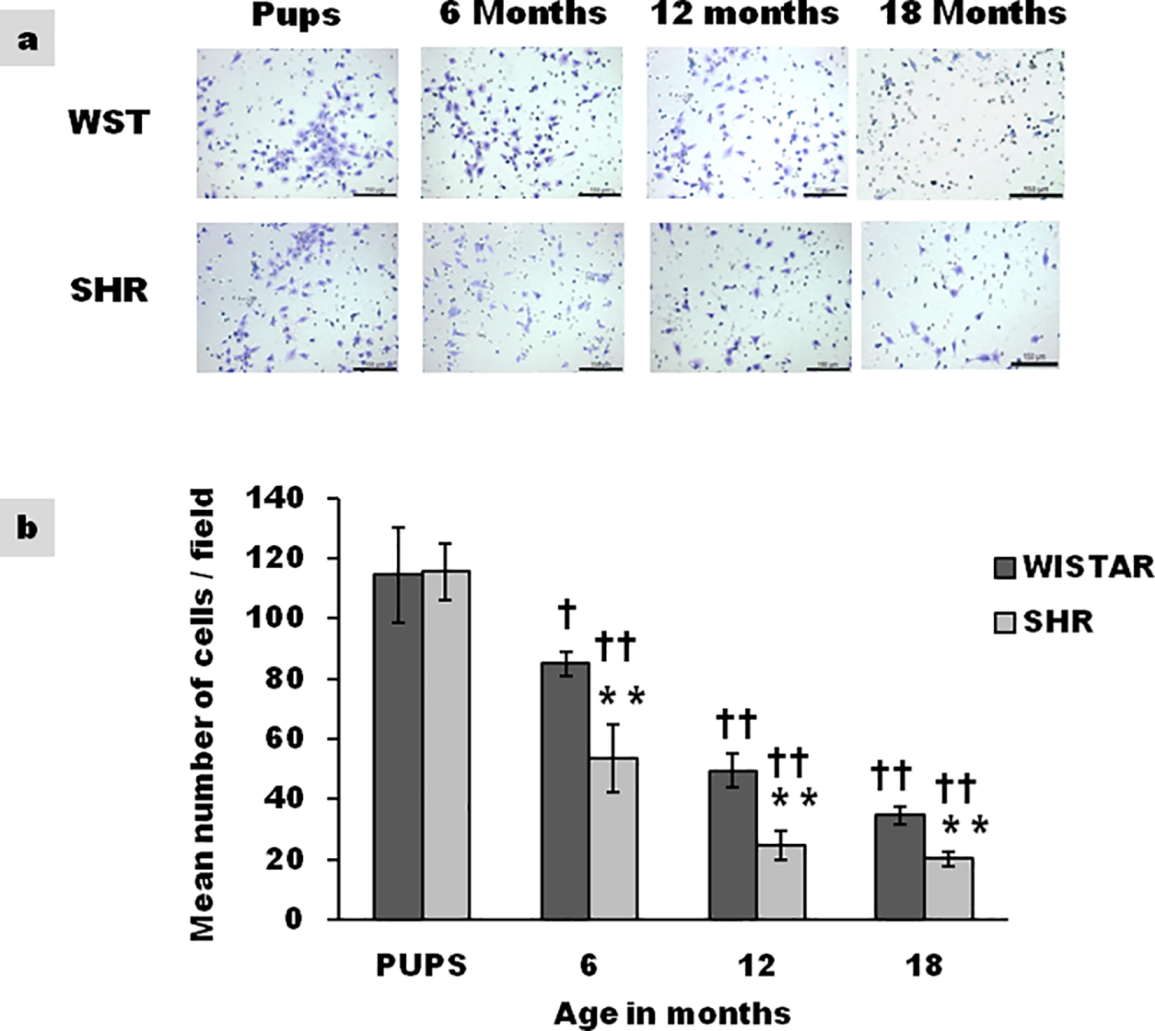
Age associated variation in migration efficiency of CSCs from WST and SHR. **(a)** Representative photograph of migrated CSCs as observed by trans-well assay. **(b)** Graphical representation of migration ability represented as mean number of cells/field (n = 3) Data presented as mean ± SD. Variation was analysed by two way ANOVA followed by Student t-test. (** p<0.01 SHR Vs Age matched WST; †† p<0.01 and † p<0.05 WST & SHR of different ages compared to respective 1 week old pups. Two way ANOVA p<0.001).

The ability of CSCs to migrate was assessed by trans-well migration assay using serum as the chemoattractant. The migratory capacity of CSCs decreased with age (Fig 3A and 3B). Significant difference in migratory capacity between SHR and Wistar was observed as early as 6 months. Stem cell migration for WST rats and SHR at 18 months was 30% and 17% respectively of that seen in pups.

### ROS levels in cultured CSCs

**Fig 4:**
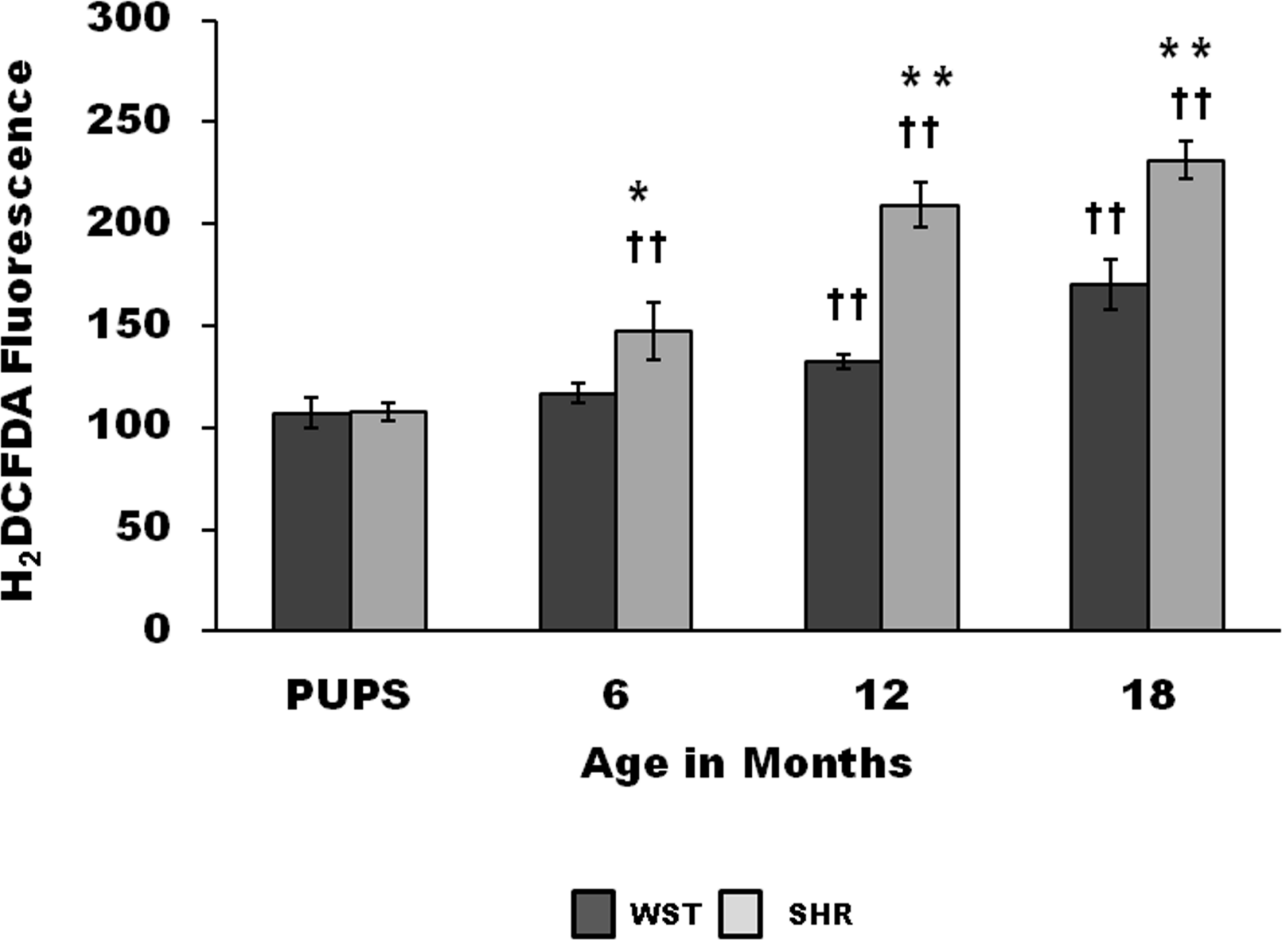
Age associated variation in ROS levels of CSCs of WST and SHR based on H2DCFDA fluorescence. Data presented as mean ± SD. (n = 3) Variation was analysed by two-way ANOVA followed by Student t-test. (**p<0.01 and * p<0.05 SHR Vs age matched WST; and †† p<0.01 WST & SHR of different ages compared to respective 1 week old pups. Two way ANOVA p<0.001).

CSCs from older rat showed a significant increase in ROS levels compared to pups (Fig 4). The ROS levels in CSCs from 6, 12 and 18-month-old SHR was significantly higher compared to age matched WST rats.

### Directed differentiation of CSCs to cardiac and vascular lineage

**Fig 5:**
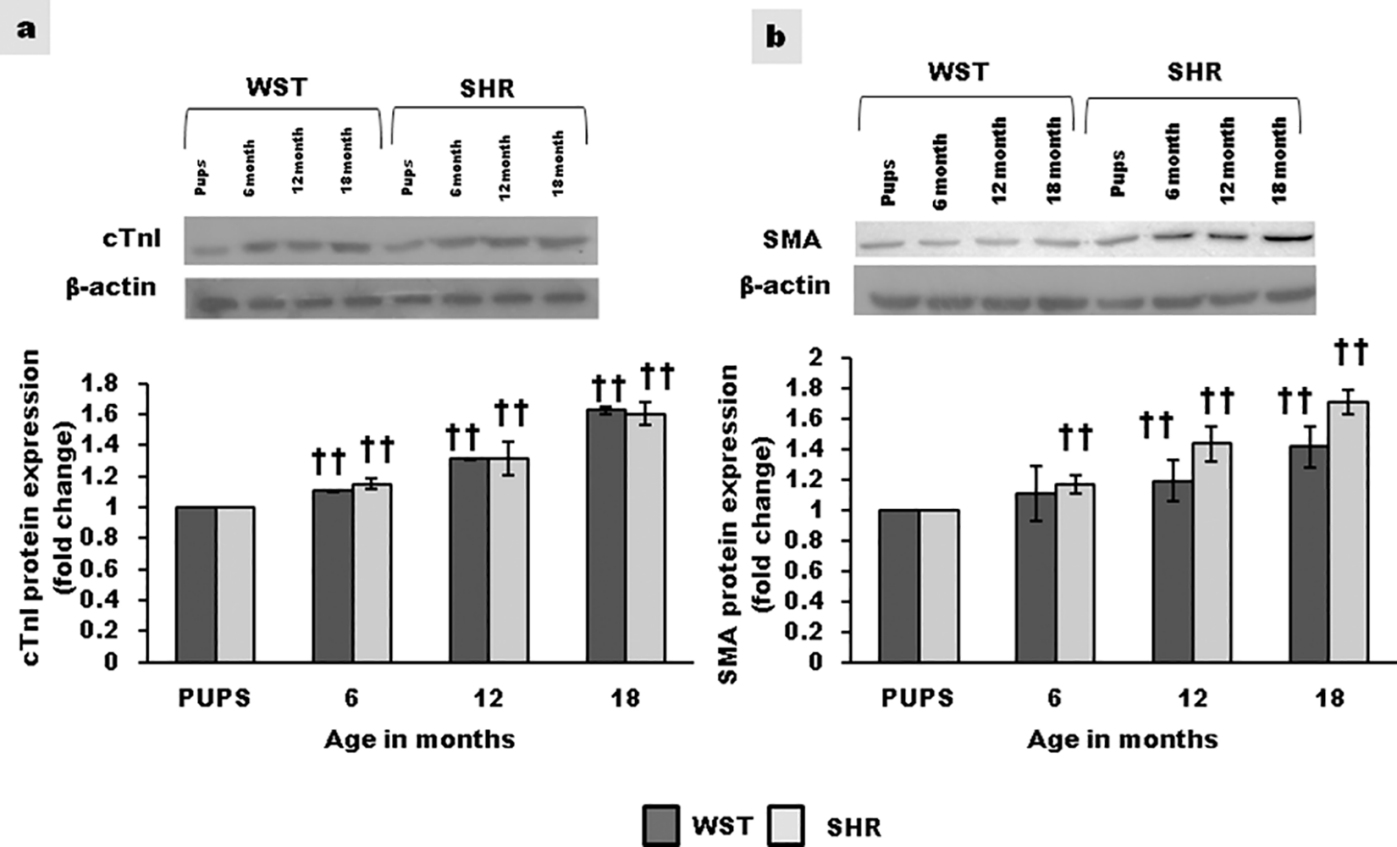
**Age associated variation in cardiovascular differentiation of CSCs from WST and SHR determined by Western blot analysis** Representative blots and graphical representation of the expression of **(a)** Cardiac troponin I and **(b)** smooth muscle actin (n = 3) Data presented as mean ± SD. Variation was analysed by two way ANOVA followed by Student t-test. (†† p<0.01 and † p<0.05 WST & SHR of different ages compared to respective 1 week old pups. Two way ANOVA p<0.001).

Expression of cardiomyocyte specific marker, cardiac troponin I and smooth muscle actin increased with age, both in WST and SHR (Fig 5). This indicates age associated tendency for lineage commitment. No significant difference was observed between SHR and WST.

### Proportion of senescent CSCs

**Fig 6:**
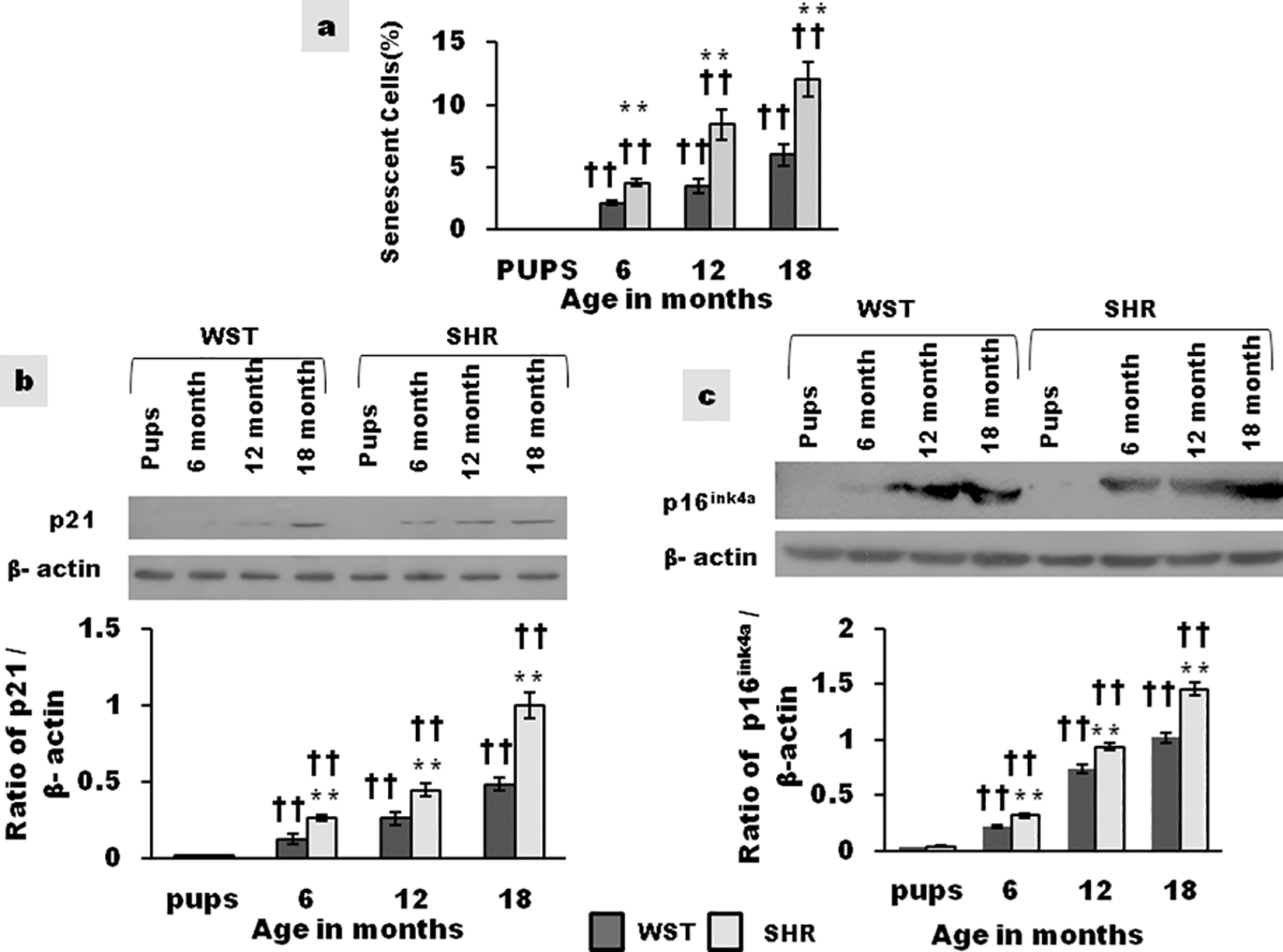
Age associated variation in the proportion of senescent CSCs in WST and SHR. **(a)** Graphical representation of percentage of senescent cells (n = 3) **(b)** Representative blots and graphical representation of p16^ink4a^ protein expression (n = 3) **(c)** Representative blots and graphical representation of p21 protein expression (n = 3) Data presented as mean ± SD. Variation was analysed by two way ANOVA followed by Student t-test. **p<0.01 SHR Vs Age matched WST; †† p<0.01 and WST & SHR of different ages compared to respective 1 week old pups. Two way ANOVA p<0.001).

The proportion of β-gal positive CSCs in 18 and 12 month old SHR was double that of age matched Wistar, indicating accelerated aging of CSCs in SHR (Fig 6A). The expression of p21 and p16^ink4a^ increased with age in both strains of rat (Fig 6B and 6C), with the expression being significantly higher in SHR compared to WST of the same age. The same pattern of expression was seen for all the three variables, testifying the age associated increase in senescence, the increase being significantly higher in SHR.

### Expression of TERT mRNA in cultured CSCs

**Fig 7:**
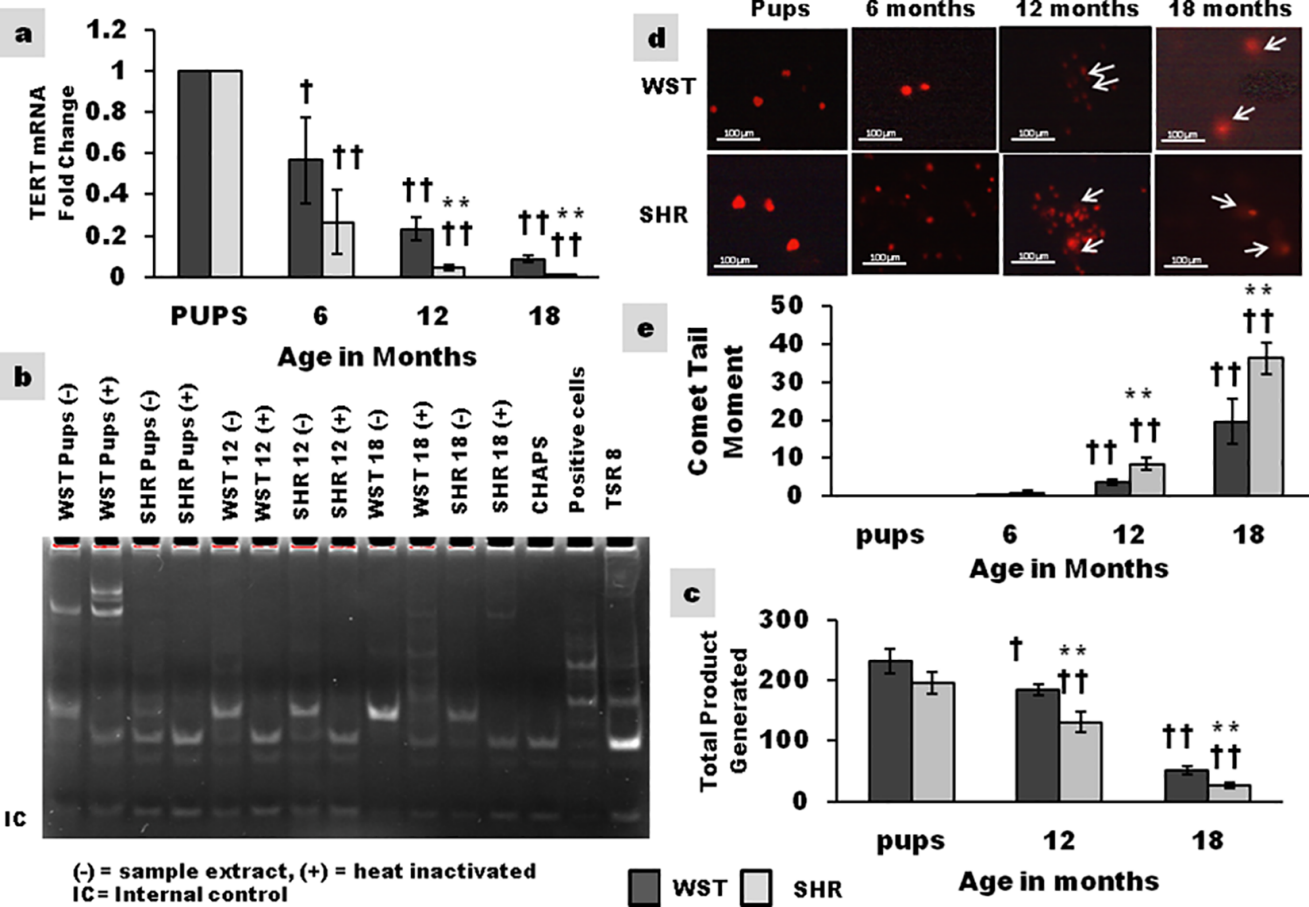
Age associated variation in expression of TERT mRNA, telomerase activity and level of DNA damage in SHR and Wistar rat. **(a)** Graphical representation of TERT mRNA levels expressed as fold change (n = 3) **(b)** Representative photograph of telomerase activity **(c)** Graphical representation of telomerase activity (n = 3) **(d)** Representative photograph of DNA damage as evaluated by Comet assay **(e)** Graphical representation of DNA damage represented as comet tail moment (n = 3) Data presented as mean ± SD. Variation was analysed by two way ANOVA followed by Student t-test. **p<0.01 SHR Vs age matched WST; †† p<0.01 and † p<0.05 WST & SHR of different ages compared to respective 1 week old pups. Two way ANOVA p<0.001).

There was a significant age associated decline in TERT expression levels in both SHR and Wistar rat (Fig 7A). SHR exhibited a highly significant decrease in TERT mRNA levels by 12 months.

### Telomerase activity in cultured CSCs

Telomerase activity was assessed by TRAP assay in 12 and 18-month-old rats and compared with the level in pups. In CSCs from 18-month-old Wistar rats the telomerase activity was 25% of that in pups and the corresponding value in SHR was 16%. The telomerase activity in CSCs from 12 and 18 month old SHR was significantly lower than that of Wistar (Fig 7B and 7C). The reduced activity of this ribonucleoprotein in cells from older animals was analogous with TERT mRNA expression.

### DNA damage in cultured CSCs by comet assay

Physiological aging resulted in 3 fold and 19 fold increases in tail moment in CSCs from 12 and 18-month-old WST rat (Fig 7D and 7E). Nevertheless, the magnitude of tail moment was about 8 fold and 36 fold in CSCs from 12 and 18-month-old SHR indicating enhanced DNA damage in hearts with pathological remodelling. As early as 6 months of age, there was significant increase in DNA damage in CSCs from SHR compared to WST.

## Discussion

Resident cardiac stem cells are vital for the maintenance of tissue homeostasis. In the event of cell loss due to tissue injury and in the process of physiological aging; stem cells home in to the affected site and mediate tissue repair. In pathological conditions, adverse microenvironment such as enhanced oxidative stress and myocyte death can also influence the properties of resident cardiac stem cells. Stem cells have been implicated in cardiac failure based on analysis of human tissues from donor and explanted failing hearts obtained at transplantation.[7] Earlier studies have shown age associated increase in myocardial oxidative stress in SHR.[23] Increased lipid peroxidation in comparison with Wistar rat was observed at 1 month of age and preceded the development of hypertrophy, highlighting the role of oxidative stress in pathologic remodelling in SHR. However, there are no reports on a systematic evaluation of cardiac stem cell characteristics in physiological aging and in a well defined pathological condition. Against this backdrop, age associated changes in the density and functional efficiency of cardiac stem cells from Spontaneously Hypertensive Rat and normotensive Wistar rat were evaluated. The study has shown that the number and efficiency of CSC declined with age, the progression being accelerated in the pathological heart.

### Age associated variation in the proportion of cardiac stem cells

Age associated decline in the density of c-kit^+^ CSCs cells was observed by FACS assay of ventricular digests (Fig 1). Uncontrolled hypertension promotes cardiac remodeling, with left ventricular hypertrophy being a leading cause for cardiac failure. Nevertheless, physiological aging is also associated with cardiac remodeling.[28] Disturbance in tissue homeostasis is a characteristic feature in cardiac remodeling. Decrease in availability of resident cardiac stem cells can adversely affect replenishment of lost myocytes for maintenance of tissue homeostasis. Repeated injury to the myocardium has been reported in left ventricular hypertrophy [9] and hypertension.[29] Therefore, in natural aging and left ventricular hypertrophy, utilization of resident cardiac stem cells for replacement of lost myocytes can result in a steady decrease in stem cell number initiated from the stage of compensatory hypertrophy. Supporting the observations of this study, Cesselli et al also reported that the number of c-kit^+^, lineage-negative CSCs was 3.2-fold higher in atria of normal donor hearts than that of diseased explanted hearts in humans as determined by Immunohistochemistry [7]. However, in the study of Cessilli et al, the age of the donor hearts being lower than the diseased heart, it is difficult to categorically state whether the decline in stem cell number is mediated by age or disease. An age and gender matched comparison of normal and diseased heart is imperative and feasible only in experimental models. The present study has shown conclusively that the age associated decline in the density of CSCs is accelerated in the diseased heart. Apart from repeated cycling of CSCs to generate new cells, the adverse microenvironment in the myocardial tissue in the form of oxidative stress can affect the characteristics of these cells, leading to stem cell aging.

### Age associated variation in stem cell characteristics

Cardiac stem cell niches are located predominantly in the atria, a region that experiences low hemodynamic stress [30] and are known to be the major source of stem cells for tissue repair. In parallel with ventricular remodeling, atrial remodeling has been reported in chronic hypertension. [29,30] As atria are the major source of stem cells, variation in stem cell characteristics with age and disease was studied in c-kit^+^ cells cultured from atrial explants. Immunomagnetic isolation of cells from atrial explant cultures yielded more than 90% lineage negative c-kit^+^ cells which were able to retain their characteristics, even upto the 10^th^ passage, as evident from the expression of surface markers (S1 Fig). Absence of hematopoietic and endothelial cell contamination was apparent from the absence of CD45 and CD31 (S1 Fig). Earlier studies have also reported the feasibility of isolating CSCs from small samples of myocardium and expansion in culture. [24]

CSCs from the atria of Wistar and SHR, of all ages could be expanded in culture to get sufficient numbers for the evaluation of stem cell characteristics. Cells in Passage 3, in all age groups were c-kit^+^ and expressed clonogenic efficiency reiterating that the sample used for the experiments were stem cells.

### Stem cell proliferation

Self renewal is an essential property for the maintenance of stem cell density. Assessment of colony forming units (CFU) (Fig 2A) demonstrates that self renewal capacity of CSCs decreases with age and that the decline occurs at an accelerated pace in SHR. The compromised proliferative capacity can be the consequence of inherent changes in the CSCs. CSCs from SHR exhibited lower growth kinetics than CSCs of WST, as apparent from decreased growth rate and increased population doubling time (Fig 2B and 2D). As early as 6-months of age, CSCs from SHR exhibited a significant increase in population doubling time and decrease in growth rate, compared to Wistar, indicating a decline in growth kinetics even during the stage of adaptive remodeling.

### Migration of cardiac stem cells

In the event of tissue damage, stem cells home in to the site of injury and mediate tissue repair, by either autocrine or paracrine mechanisms, [31] Migration is therefore vital for efficient tissue repair. During an insult, activated stem cells navigate from the atria to the ventricles, since majority of CSCs of the heart reside in atria.[32] Age associated decrease in migratory capacity (Fig 3) can affect efficient tissue repair. CSCs from SHR were found to have lower migratory capacity on comparison with age matched WST implying compromised efficiency. Though no information is available on CSCs, age associated decrease in functional ability of hematopoietic stem cells has been reported.[33]

### Oxidative stress in cardiac stem cells

Increase in oxidative stress is linked with aging. Excessive amounts of ROS is reported to be the cause for cellular senescence, apoptosis and carcinogenicity.[34] Hematopoietic stem cells, neuronal stem cells and early progenitors[35] contain lower levels of ROS than their mature progeny, and these differences are said to be critical for maintaining stem cell function.[36] Li et al reported that physiological levels of intracellular ROS are required to maintain genomic stability in cardiac and embryonic stem cells through activation of the DNA repair pathway.[37] Presence of optimal oxidant status in the microenvironment is therefore essential for maintaining a pool of healthy stem cells. Relatively higher ROS level was observed in the CSCs of SHR (Fig 4). The increased ROS level in CSCs of SHR is possibly the consequence of the suboptimal extracellular milieu. Increased myocardial oxidative stress compared to Wistar rat was seen as early as 4 weeks of age in SHR.[23] This can be one of the causative factors for the higher oxidative stress in stem cells, which in turn can influence the functional characteristics of stem cells. Antihypertensive drugs that reduce oxidative stress were found to restore stem cell efficiency. (Unpublished observation)

### Directed differentiation of cardiac stem cells to cardiovascular lineage

On stimulation with 5-azacytidine, the expression of myocyte specific marker, cardiac Troponin I and vascular smooth muscle actin increased with age both in SHR and WST (Fig 5). ROS is required for differentiation into cardiomyocytes from embryonic, haematopoietic and cardiac stem cells.[38–40] Decrease in proliferative potential associated with the presence of oxidative stress can promote lineage commitment. Tendency for unequal division with, lineage commitment is known to increase with age.[41] This in turn can lead to reduction in the proportion of stem cells. The presence of dividing myocytes has been reported in aged as well as end-stage cardiac failure patients.[42] Hence, the increase in differentiation potential upon stimulation reflects the inherent property of the CSCs of the aged heart with lineage commitment to form new blood vessels and myocytes.

### Aging of cardiac stem cells

The expression of senescence-associated markers p21 and p16^ink4a^ and the proportion of SA-β-gal positive cells increased with age (Fig 6). The proportion of senescent cells was significantly higher in SHR compared to age matched Wistar rat (Fig 6). Senescence and death of CSCs with increasing age in wild type mice has been implicated in impairment of growth and turnover of cells in the heart.[25] Senescent stem cells affect their microenvironment by decreasing regenerative potential of the entire stem cell pool, while also affecting neighboring myocytes and vasculature.[43] This study for the first time reports the increased expression of p16^ink4a^ and p21 in CSCs with age and its preponderance in SHR. The difference between SHR and Wistar was apparent as early as 6 months of age, which is the compensatory phase of hypertrophy. Tissue necrosis and myocyte death in 6 month old SHR has been attributed to the diminished tissue oxygenation due to decreased capillary density.[44] Paracrine factors secreted by apoptotic and necrotic myocardial cells can adversely affect the efficacy of CSCs.

Telomeres play an essential role in cellular aging and is dictated by down-regulation of telomerase, oxidative stress, and loss of telomere-related proteins.[45,46] Telomerase protects chromosomes from telomere erosion, maintains cell replication and opposes cell death.[47] The expression of TERT mRNA and the telomerase activity declined with age, and at a significantly higher rate in SHR (Fig 7). In humans with ischemic heart failure, telomerase activity declined, and CSC division was impaired by severe telomeric shortening and cellular senescence.[48] DNA damage in 12 and 18-month-old rat was significantly higher in SHR (Fig 7E). Though no information is available on the fate of CSCs, higher rate of senescence and lower rate of telomerase activity of endothelial progenitor cells from SHR compared to age matched WST has been reported.[49] Decrease in telomerase activity and enhanced DNA damage along with the expression of senescence associated markers denote accelerated aging of CSCs in SHR.

## Conclusion

Chronological age is a major determinant of the loss in growth reserve of the adult heart, dictated by progressive decline in the density and efficiency of CSCs. Age associated decrease in efficiency of stem cells can be responsible for the degenerative cardiac changes in physiological aging. Aging of CSCs can affect migration and proliferation and promote apoptosis. Accelerated aging in stem cells isolated from hearts of SHR is possibly mediated by an adverse microenvironment. This is the first study to examine the fate of cardiac stem cells in chronic pressure overload hypertrophy. Decrease in healthy stem cell pool can affect efficient tissue repair and precipitate the transition from compensated hypertrophy to cardiac failure. Enhanced oxidative stress in the microenvironment can be a predominant factor contributing to stem cell aging. The salient findings of accelerated decline in cardiac stem cell efficiency in SHR provide insight for further studies to examine whether reduction of cardiac oxidative stress can restore stem cell function and prevent progressive cardiac remodeling.

## Supporting information

S1 Fig (a) Atrial explant culture at days 4 and 18 (b) CSC culture at days 3, 6 and 12 (c) FACS images for the expression of c-kit, CD 45 and CD 34 at passage 3 (d) Immunocytochemistry for the expression of c-kit, CD 45 and CD34 at passage 3 (e) FACS image for the expression of c-kit at passage 10.
(TIF)
