# Supplementary figures and images for: Accelerated decline in cardiac stem cell efficiency in Spontaneously hypertensive rat compared to normotensive Wistar rat
Source: PLoS One. 2017 Dec 12;12(12):e0189129. doi: 10.1371/journal.pone.0189129 (PMC5726722; doi:10.1371/journal.pone.0189129)

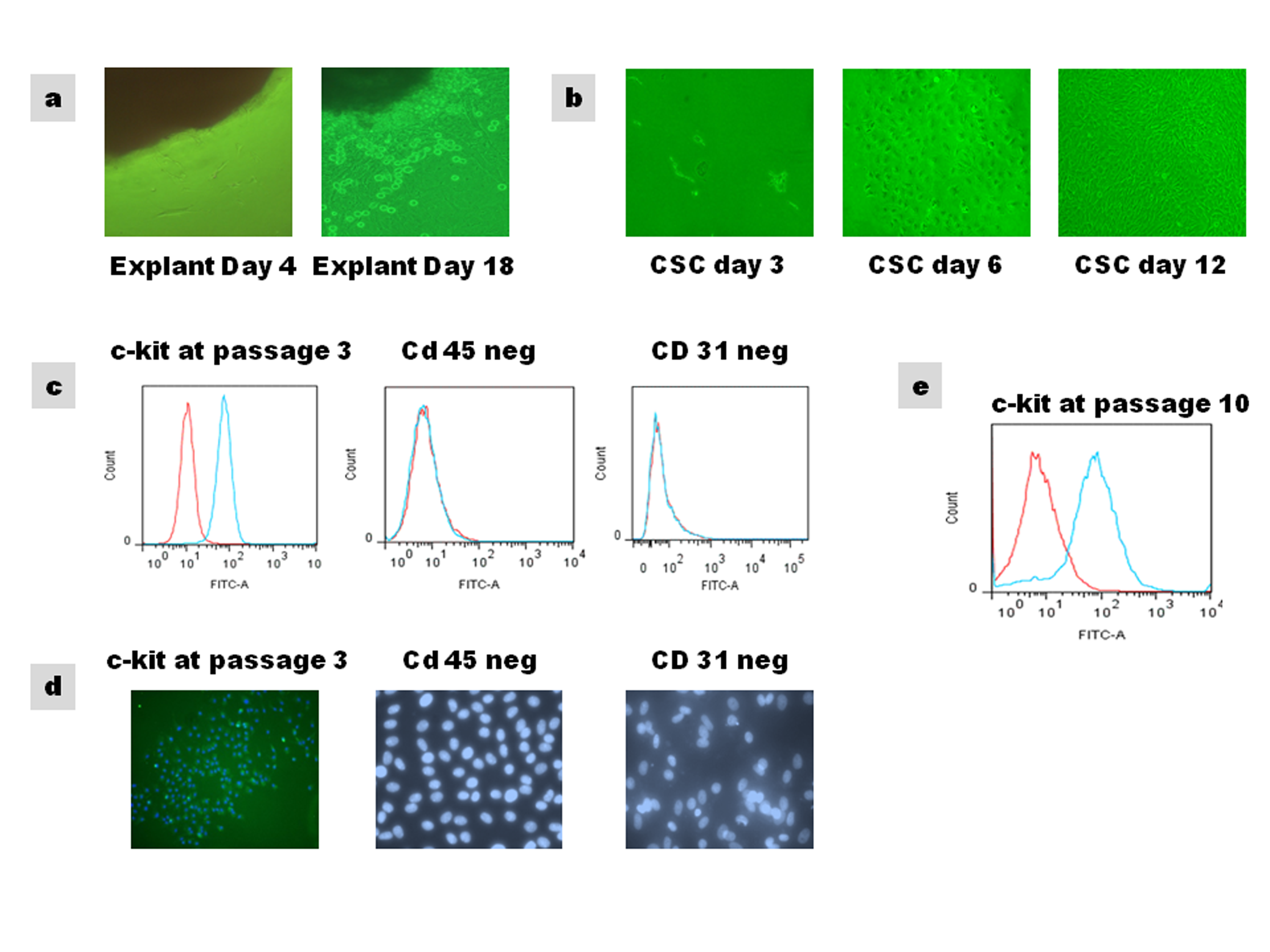

Supplement: S1 Fig — (a) Atrial explant culture at days 4 and 18 (b) CSC culture at days 3, 6 and 12 (c) FACS images for the expression of c-kit, CD 45 and CD 34 at passage 3 (d) Immunocytochemistry for the expression of c-kit, CD 45 and CD34 at passage 3 (e) FACS image for the expression of c-kit at passage 10. (TIF) [file pone.0189129.s001.tif]
